# Fluorescent Nanosensor for Indole‐3‐Propionic Acid Detection in Gut Health Monitoring

**DOI:** 10.1002/adhm.202503434

**Published:** 2026-05-02

**Authors:** Mervin Chun‐Yi Ang, Jonathan Wei Jie Lee, Sayyid Mohaideen, Gabriel Sánchez‐Velázquez, Song Wang, Yangyang Han, Liu Lin, Germaine Yong, Raju Cheerlavancha, Duc Thinh Khong, Jianhong Ching, Sharon Hong Yu Han, Jun Yu Yeo, Lian Xu, Gajendra Pratap Singh, Michael S. Strano

**Affiliations:** ^1^ Disruptive & Sustainable Technologies for Agricultural Precision IRG Singapore‐MIT Alliance for Research and Technology Singapore Singapore; ^2^ Natural Sciences and Science Education National Institute of Education Nanyang Technological University Singapore Singapore; ^3^ Yong Loo Lin School of Medicine National University of Singapore Singapore Singapore; ^4^ Division of Gastroenterology and Hepatology National University Hospital Singapore Singapore; ^5^ iHealthtech National University of Singapore Singapore Singapore; ^6^ Department of Chemical Engineering Massachusetts Institute of Technology Cambridge Massachusetts USA; ^7^ State Key Laboratory of Mesoscience and Engineering Institute of Process Engineering Chinese Academy of Sciences Beijing China; ^8^ Singapore Institute of Food and Biotechnology Innovation (SIFBI) Agency for Science Technology and Research (A*STAR) Singapore Singapore; ^9^ Cardiovascular and Metabolic Diseases Programme Duke‐National University of Singapore Graduate Medical School Singapore Singapore

**Keywords:** carbon nanotubes, conjugated polyelectrolyte, gut health, Indole‐3‐propionic acid, nanosensor

## Abstract

The gut microbiota plays a pivotal role in bio‐transforming dietary components, including tryptophan, an essential amino acid that undergoes microbial metabolism. Microbial metabolism of tryptophan yields indole‐3‐propionic acid (IPA), an emerging biomarker for gut inflammation. Current IPA detection relies on expensive, time‐consuming mass spectrometry. To address this limitation, a fluorescent nanosensor system is presented that uniquely features two optical modalities: one utilizing near‐infrared (NIR) emission of a central single‐walled carbon nanotube (SWNT), and a separate, visible emission from the corona phase polymer, a cationic conjugated polyelectrolyte (CP3). Selective IPA molecular recognition occurs at the latter, but the binding is optically reported via quenching in both the visible and NIR emission channels. The two modalities provide complementary advantages: CP3‐SWNTs’ NIR channel enables detection in strongly scattering tissue environments due to reduced Rayleigh scattering at longer wavelengths. Conversely, CP3 visible channel facilitates future rapid, cost‐effective point‐of‐care biological sample screening. Functionality of both modalities is maintained within a gelatin metacrylate hydrogel offering potential for future continuous in vivo monitoring of IPA dynamics. The sensor reveals significant differences in plasma IPA levels between healthy controls and patients with active gut inflammation: ulcerative colitis and Crohn's disease, highlighting its promise in rapid gut health assessment.

## Graphical abstract

This work introduces a fluorescent nanosensor system with two optical modalities for detection of indole‐3‐propionic acid (IPA) gut metabolite: (1) blue‐emitting cationic conjugated polyelectrolyte (CP3) and (2) near‐infrared (NIR)‐emitting CP3‐wrapped single‐walled carbon nanotube (SWNT). The sensor can provide clear distinction of IPA levels across healthy controls, inflammatory bowel disease and colorectal cancer cohorts, demonstrating promise for rapid gut health assessment.

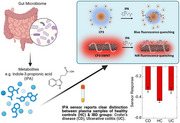

## 1 Introduction

The diverse microbial community in the human gut converts dietary substrates, such as tryptophan (Trp), into bioactive metabolites through a variety of enzymatic pathways, including the indole, kynurenine and serotonergic pathways [1, 2, 3, 4]. Among these, the indole pathway represents a major route for gut microbial metabolism of Trp, yielding indole derivatives, such as indole‐3‐propionic acid (IPA) [5]. In recent years, IPA has garnered significant research interest due to its vital role in maintaining gut health and its influence on systemic physiological processes beyond the gastrointestinal system [6]. IPA exhibits anti‐inflammatory and antioxidant properties by scavenging hydroxyl radicals, thereby reducing oxidative stress and lipid peroxidation [7, 8, 9]. Notably, selective IPA deficiencies have been observed in serum of human subjects with active ulcerative colitis, with levels normalizing upon remission, highlighting IPA as a potential biomarker for both active ulcerative colitis and its remission [10]. Emerging evidence also suggests IPA as a promising biomarker for the onset and development of metabolic disorders, including type 2 diabetes [11, 12, 13] and non‐alcoholic fatty liver disease [12, 14], potentially paving the way for novel preventive and therapeutic strategies.

Given the increasing recognition of IPA's importance in gut health and disease, accessible advancements in its detection and quantification are critical. To date, IPA analysis primarily relies on conventional mass spectrometry‐based techniques [15, 16] which are expensive and time‐consuming, rendering them non‐ideal for routine testing of emerging biomarkers like IPA. Recent sensor developments for indole‐based metabolites have targeted related metabolites such as indole, tryptamine, indoxyl sulfate, tryptophan or indole acetic acid (IAA), using either electrochemical or optical approaches [17, 18, 19, 20, 21]. For instance, a nanotip array‐based electrochemical sensor platform was developed recently for detection of indole, tryptamine and indoxyl sulfate [21]. Optical and electrochemical probes for IAA, tryptophan and tryptamine have also been developed for real‐time and continuous measurement [17, 18, 19, 20]. In contrast, no optical or electrochemical sensors have been reported for specific detection of IPA. Hence, reliable and rapid sensing technologies are urgently needed to understand the dynamics of IPA levels in the human gut, in order to provide early insights into gut health status and potentiate timely interventions through diet or microbial modulation.

**FIGURE 1:**
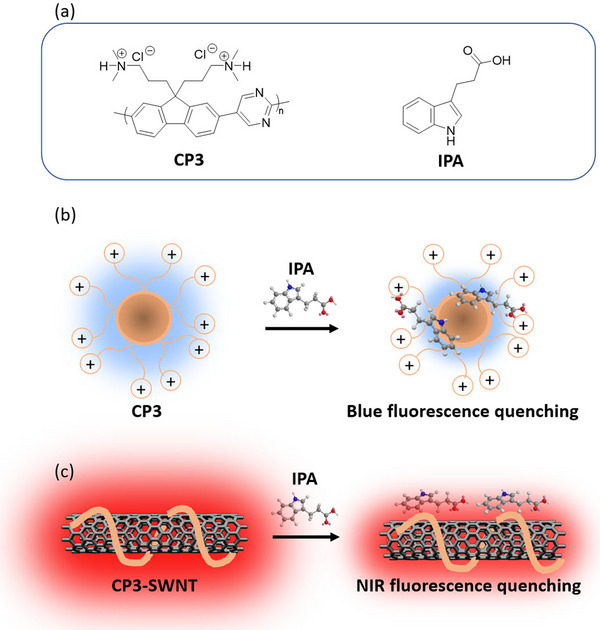
Development of two spectrally distinct fluorescent nanosensors for IPA detection. (a) Chemical structures of CP3 polymer (left), which comprises a fluorene monomer with tethered dimethylammonium cations, co‐polymerized with a pyrimidine monomer, and IPA bio‐analyte (right); (b) Schematic of CP3 polymeric micelle, which exhibit blue fluorescence quenching upon binding of IPA. (c) Schematic of a CP3 polymer wrapped SWNT, which exhibits NIR fluorescence quenching upon binding of IPA.

To address these limitations, we have developed an optical nanosensor for IPA that has two fluorescent emission channels (visible and near‐infrared) (Figure 1a). It is based on conjugated polyelectrolyte nanoparticles, which serves as the nanoparticle corona, and their non‐covalent functionalization of single‐walled carbon nanotubes (SWNTs). Conjugated polymers are a class of optically active materials increasingly used for sensing. Their extended π‐electron systems enable signal amplification through efficient exciton migration. Compared to small fluorophores, they exhibit larger absorption extinction coefficients and more efficient energy transfer, allowing for greater sensitivity and signal output [22, 23]. Conjugated polyelectrolytes offer further benefits for biosensing with their water solubility and ability to form stable assemblies with bio‐analytes [24, 25, 26]. For the IPA sensor, we utilized a cationic conjugated polyelectrolyte (CP3) that self‐assembles in aqueous solution to form blue‐fluorescent polymeric micelles [27, 28]. The blue fluorescence from the CP3 polymeric micelles is selectively quenched upon IPA binding (Figure 1b), as IPA induces an increase in their micellar number.

CP3 also effectively wraps SWNTs, forming stable near‐infrared (NIR)‐fluorescent nanoparticles. SWNTs have emerged as promising nanosensors, due to their unique optical properties [29]. Corona Phase Molecular Recognition (CoPhMoRe) is a technique that enhances SWNT selectivity by creating synthetic molecular recognition sites [30] through non‐covalent wrapping with different amphiphilic heteropolymers [31, 32]. Diverse libraries of CoPhMoRe‐based sensors have been developed and utilized for recognition of biomedical analytes, including nitric oxide [33], hydrogen peroxide [34], neurotransmitters [35], steroids [36] and cytokines [37]. This binding event triggers significant NIR fluorescence intensity change of the SWNTs, providing a detectable optical signal, while remaining unresponsive to other interfering molecules. This work expands the success of the CoPhMoRe platform to a NIR‐active IPA nanosensor that exhibits fluorescence quenching upon IPA binding (Figure 1c). Uniquely, we have found that the CP3 polymer is capable of energy transfer to SWNT. We have leveraged both the blue fluorescence quenching of CP3 nanoparticles and the NIR fluorescence quenching of CP3‐wrapped SWNTs to create two spectrally distinct optical nanosensors for IPA detection. The NIR fluorescence of the CP3‐SWNT channel is well‐suited for potential in vivo monitoring of IPA dynamics, due to the ability to probe deeper tissues up to 3 cm with minimal tissue autofluorescence and scattering [38, 39, 40, 41]. Conversely, the blue fluorescence of the CP3 nanoparticles facilitates rapid and inexpensive screening of biological fluid samples from clinical subjects, without the need for complex NIR‐sensitive optical components [42]. Leveraging the visible channel of the nanosensor, we have successfully demonstrated its ability to detect significant differences in IPA levels in plasma samples obtained from healthy individuals and participants with intestinal diseases (i.e. ulcerative colitis and Crohn's disease). This highlights the promising applicability of our sensor for rapid and accessible gut health assessment.

## 2 Results and Discussion

### 2.1 CP3‐SWNT Synthesis and Characterization

**FIGURE 2:**
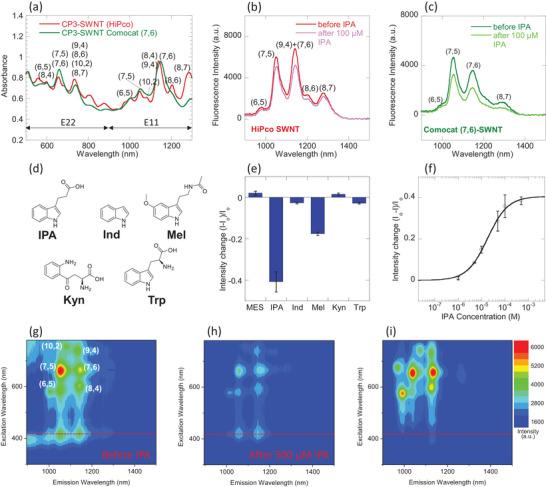
NIR fluorescent IPA sensor based on CP3‐wrapped SWNT. (a) UV–Vis‐NIR absorption spectra of HiPco‐SWNT and Comocat (7,6)‐enriched SWNT wrapped with CP3 after 2 h ultracentrifugation at 153100 g. E11 and E22 absorption bands of different SWNT chiralities are labelled in parenthesis; NIR fluorescence spectra of (b) HiPco SWNT and (c) Comocat (7,6)‐enriched SWNT wrapped with CP3 polymer, before and after mixing with 100 µM IPA, which shows significant fluorescence quenching response. (d) Chemical structures and abbreviations of gut metabolites screened; (e) Sensor responses to 100 µM gut metabolites which show most significant response to IPA binding. Error bars represent standard deviations from independent experiments (n = 3). Blank MES buffer (10 mM, pH 5.5) is used as negative control; (f) Calibration curve of CP3‐SWNT against IPA concentration which determines K_D_ of 18 µM (R = 0.998). Error bars represent standard deviations from independent experiments (n = 3); 2D excitation‐emission map of CP3‐SWNT (g) before and (h) after addition of IPA, as well as (i) (GT)_15_‐SWNT. Red dotted line indicates the SWNT photoluminescence arising from energy transfer from CP3 polymer to SWNT. (GT)_15_‐SWNT does not have SWNT photoluminescence at this excitation wavelength.

Building on our previous work on developing conjugated polyelectrolyte‐wrapped SWNT nanosensors for plant phytohormones, such as 2,4‐dichlorophenoxyacetic acid (2,4‐D) [15] and salicylic acid (SA) [32], we employ in this study a similar cationic fluorene monomer in the IPA sensor development. Here, the cationic fluorene monomer is co‐polymerized with a pyrimidine monomer in CP3 (Figure S1), in contrast to the phenyl and pyrazine monomers used in the 2,4‐D and SA sensors respectively. The aromatic polymer backbone of CP3 facilitates strong π‐π interactions with the SWNT surface upon ultrasonication, leading to formation of stable aqueous SWNT suspensions essential for sensor applications. After 2 h of ultra‐centrifugation, CP3‐SWNT suspensions of 90–100 mg/L were obtained (Figure 2a). Initial experiments with HiPco SWNTs, a mixture of SWNT chiralities including (6,5), (8,4), (7,5), (10,2), (9,4), (7,6), (8,6) and (8,7), revealed a chirality‐dependent quenching response upon IPA addition (Figure 2b). For instance, the intensity of (6,5) SWNT peak was relatively unchanged upon IPA addition, whereas the combined (9,4) + (7,6) peak showed the largest quenching response. To enhance the overall quenching response, we switched to Comocat (7,6)‐enriched SWNTs (Figure 2c). This enrichment in the responsive (7,6) chirality led to a ∼75% increase in magnitude of IPA sensor response compared to HiPco SWNTs, thus improving sensor sensitivity.

The hydrodynamic radius distribution of CP3‐SWNT, measured using single particle tracking analysis based on the maximum a posteriori nanoparticle tracking analysis (MApNTA) algorithm [43, 44], shows a unimodal distribution with the dominant mode at *R_h_* =  43 *nm* (Figure S2) [43, 45]. To estimate the thickness of the polymer corona (α) around the nanotube, we have approximated the volume of CP3‐SWNT cylinder (π(*r_t_* + α)^2^ *L*) to the volume of a spherical nanoparticle $\left(\frac{4}{3}\pi {R_{h}}^{3}\right)$. Assuming nanotube length *L*  ≈ 300 *nm* and a Comocat (7,6)‐SWNT core radius *r_t_*  ≈ 0.42 *nm*, CP3 corona thickness is derived to be α  ≈ 18.4 *nm*. This estimated thickness is significantly larger than other corona phases, such as surfactant (SDS‐SWNT ∼2.8 nm), DNA (s.s. (GT)_15_‐SWNT ∼3.8 nm) or polymer (RITC‐PEG‐RITC‐SWNT ∼8.1 nm) [30, 43], suggesting that there is potentially CP3 nanoparticle assembly on the SWNT surface.

### 2.2 CP3‐SWNT Sensor Selectivity and Sensitivity

To evaluate the selectivity of CP3‐wrapped (7,6) SWNT, we assessed its response to a panel of relevant gut metabolites and precursors (Figure 2d), including compounds with a high degree of structural similarities to IPA and compounds known to be altered under gut inflammation conditions. These analytes include: indole (Ind), melatonin (Mel), kynurenine (Kyn) and tryptophan (Trp). Remarkably, CP3‐SWNT shows a highly selective quenching response to IPA (41%), while remaining relatively inert to Ind, Kyn and Trp (Figure 2e). It also shows a moderate quenching response to Mel (18%). The selective response to IPA is mediated by the assembly of the CP3 corona onto the SWNT surface that creates a specific binding site for IPA, characteristic of CoPhMoRe sensors. As the hydrophobic CP3 polymer backbone adsorbs onto the SWNT surface, the hydrophilic cationic side chains extend out into the solution, forming a positively charged binding interface. Hence, we attribute the selective response to IPA to the strong electrostatic interactions between the cationic ammonium side chains of the polymer and the carboxylate group of IPA, combined with π−π interactions with the indole ring and the CP3 backbone. Comparatively, the Ind molecule lacks an anionic group for electrostatic interactions. Trp and Kyn, both amino acids, could exist as zwitterions at physiological pH. Hence, they are also likely to exhibit reduced electrostatic attraction to the cationic CP3 polymer, due to the presence of an intramolecular positive charge. The moderate response of CP3 to Mel could arise from weak interactions between the partial negative charge on the oxygen atoms of the acetylamide and ether groups with the cationic CP3. We also assessed that CP3‐SWNT has preferential binding to IPA, even in the presence of these gut analytes (Figure S3a). It was observed that even in the presence of interferent molecules that gave a moderate quenching response, subsequent addition of IPA triggers a strong, consistent quenching response, similar to the response observed when IPA is added alone.

In previous work, the IPA sensor is found to be inert to key plant phytohormones including indole acetic acid (IAA) and indole butyric acid (IBA) [31], which are also gut metabolites. They have the closest chemical structural resemblance to IPA, differing only by the length of their alkyl side chains. The lack of sensor binding to IAA or IBA could be attributed to the difference in chain length, which hindered optimal spatial fitting within the CP3 polymer corona. Taken together, these results indicate that CP3‐SWNT exhibits a relatively selective response for the IPA analyte. Furthermore, a similar quenching response has been observed when the neutral IPA sodium salt (IPA‐Na) was added (Figure S3b), confirming that the sensing mechanism is driven by specific electrostatic binding of IPA, and is not a non‐specific pH effect.

To quantify the binding strength of CP3‐SWNT to IPA, we performed a titration of CP3‐SWNT against IPA concentrations ranging from 1 µM to 500 µM (Figure S3c). With increasing IPA concentration, intensity of fluorescence quenching increases. Fluorescence quenching intensity ($\frac{I-I_{o}}{I_{o}}$) was calculated upon addition of each IPA concentration (Figure 2f). The data is fitted to the Langmuir adsorption model: $\frac{I-I_{o}}{I_{o}}=-\beta \frac{\left[IPA\right]}{K_{D}+\left[IPA\right]}$, where β is a proportional constant corresponding to the maximum optical modulation under sensor saturation conditions, and K_D_ is the dissociation constant, which measures the binding affinity between IPA and CP3‐SWNT. Here, the K_D_ of the IPA‐CP3‐SWNT complex is 18 µM. The sensor limit of detection (LOD) for IPA, defined as the concentration required to achieve a signal‐to‐noise ratio of ≥3, was calculated to be 4.1 µM. 2D excitation‐emission maps of CP3‐SWNT before (Figure 2g) and after (Figure 2h) IPA addition revealed significant fluorescence quenching across a broad range of excitation wavelengths (300–780 nm). Consistent with observations in other polyfluorene wrapped SWNT systems [46, 47], strong SWNT fluorescence was observed upon excitation at 380 – 420 nm, coinciding with the CP3 polymer absorption band. The spectral overlap between CP3 emission and SWNT E33 absorbance bands (Figure S3d), along with the observed enhancement of SWNT fluorescence upon excitation of the CP3 polymer, suggests efficient Förster Resonance Energy Transfer (FRET)‐like energy transfer from the polymer to the SWNTs, leading to exciton generation and subsequent NIR emission. Comparatively, the 2D excitation‐emission map of a single‐stranded oligonucleotide (GT)_15_‐wrapped SWNT (Figure 2i) shows the absence of SWNT fluorescence at 380–420 nm, as (GT)_15_ is incapable of photoexcitation at these wavelengths and energy transfer to SWNTs.

To assess the compactness of the CP3 corona phase around the SWNTs, we used the molecular probe adsorption (MPA) method [48, 49]. MPA quantifies the accessible SWNT surface area by measuring the adsorption of a quenchable fluorescent dye, such as riboflavin, onto the SWNT surface. By titrating CP3‐SWNT with increasing concentrations of riboflavin (0–5 µM) and measuring the riboflavin fluorescence at each point, we can determine the extent of fluorescence quenching (Figure S4a). This quenching is proportional to the amount of riboflavin adsorbed, and consequently, the accessible SWNT surface area. The key parameter derived from MPA is $\frac{q}{K_{D}}$, where (*q*) represents the accessible surface area, and *K_D_* is the dissociation constant for riboflavin binding (Figure S4b). A smaller $\frac{q}{K_{D}}$ corresponds to a smaller accessible SWNT surface area, indicating a more densely packed corona which blocks adsorption of the probe. Detailed MPA derivation of $\frac{q}{K_{D}}$ is described in Supplementary Text. The relatively low $\frac{q}{K_{D}}$ of 307 M^−1^ observed for CP3‐SWNT, indicating a crowded corona on the nanotube surface, can be attributed to CP3 polymer's rigid, rod‐like conjugated polymer backbone [50], which facilitates strong inter‐chain π‐π interactions, enabling closer packing on the SWNT surface. Comparatively, DNA‐wrapped SWNTs ($\frac{q}{K_{D}}$ = 400–800 M^−1^) and non‐conjugated polymers such as poly(sodium 4‐styrene sulfonate), dextran and chitosan ($\frac{q}{K_{D}}$ = 1000–3000 M^−1^) have significantly higher $\frac{q}{K_{D}}$ values, indicating a more open, solvent exposed nanotube surface [48]. This result also implies a shorter average distance between CP3 polymer and SWNT. Given that short‐range energy transfer processes such as FRET tend to be highly distance‐dependent [51], the observed energy transfer between CP3 and SWNT could potentially be influenced by this densely packed corona. While CP3‐SWNT has shown photophysical behavior consistent with FRET, the IPA sensor measurements have utilized excitation wavelengths that target SWNT absorbance directly to provide a more reliable and direct correlation between analyte interaction and SWNT emission.

### 2.3 CP3 Polymer Sensor Selectivity and Sensitivity

**FIGURE 3:**
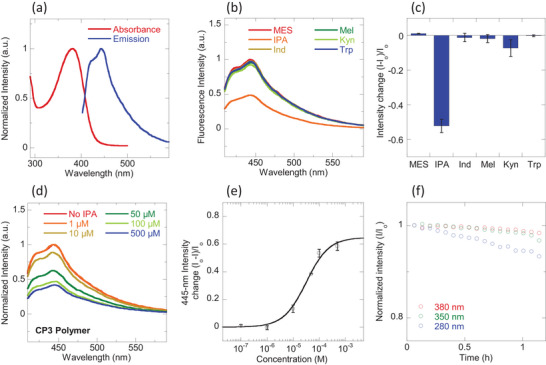
Blue‐fluorescent IPA sensor based on CP3 polymer. (a) Normalized optical absorbance and emission spectra of bare CP3 polymer without SWNT; (b) Normalized emission spectra of CP3 polymer upon binding to different gut metabolites (100 µM) including IPA; (c) Sensor responses to gut metabolites which show highly selective response to IPA binding. Error bars represent standard deviations from independent experiments (n = 3); (d) Normalized emission spectra of CP3 polymer upon addition of different concentrations of IPA from 1–500 µM; (e) Calibration curve correlating intensity change of 445‐nm λ_em_ peak of CP3 polymer emission spectra to IPA concentration, which gives sensor K_D_ of 32 µM (R = 0.996). Error bars represent standard deviations from independent experiments (n = 3); (f) Demonstration of CP3 optical stability to constant exposure of UV light at three different wavelengths of 280 nm (blue), 350 nm (green) and 380 nm (red) for up to 1 h.

Fluorene‐based conjugated polyelectrolytes have been used in detecting various biomolecules [52], as exemplified by their recent applications for adenosine triphosphate (ATP) [53] and dopamine [54] detection. To establish a baseline for comparison with CP3‐SWNT sensor, we first characterized the photophysical properties of CP3 alone, in the absence of SWNTs. Polyfluorenes generally absorb in the UV region (360–400 nm) and emit blue light (420–480 nm) [55]. Consistent with these fluorene‐based polymers, CP3 exhibited a maximum excitation wavelength (λ_ex_) at 383 nm and a maximum emission wavelength (λ_em_) at 445 nm (Figure 3a). We then evaluated the selectivity of CP3 for IPA by measuring its fluorescence response to the same panel of gut metabolites used in the SWNT experiments. Remarkably, CP3 showed negligible response to all metabolites except IPA (Figure 3b), which elicited a significant 52% fluorescence quenching (Figure 3c). The enhanced selectivity of the CP3 polymer itself is notable, especially when compared to the CP3‐SWNT sensor, which had displayed a moderate quenching response upon addition of Mel. Note that in CoPhMore, it is not usually the case that the polymer itself, independent of adsorption on the cylindrical nanotube, show analyte selectivity [56, 57]. In this case, the free fluorene co‐polymer itself, with its blue‐fluorescent backbone, turns out to be a superior sensor for this particular analyte detection problem. However, the selectivity difference between the CoPhMoRe phase (i.e. on the nanotube) and the free polymer sensor is a clear example of how the curvature and other properties of the nanotube induce a unique molecular binding site, distinct from the free polymer itself.

In the absence of SWNTs, the amphiphilic CP3 polymer self‐assembles into polymeric micelles in aqueous solution, as supported by scanning electron microscopy (Figure S5a) and dynamic light scattering (DLS) measurements. DLS measurements revealed a bimodal size distribution (Figure S5b), indicative of two distinct micelle populations: (1) primary micelles with hydrodynamic diameter peak at 32.7 nm and (2) micellar aggregates with hydrodynamic diameter peak at 164 nm. When complexed with SWNTs, we have estimated CP3 corona thickness to be α ≈ 18.4 nm, which is comparable to the radius of a CP3 primary micelle (16.35 nm). This supports our postulation that the CP3 has formed self‐assembled nanoparticles around the SWNT surface. In the absence of SWNTs, the CP3 micellar aggregate peak is more prominent than the primary micelle peak, indicative of a preference for higher‐order micellar assemblies. The larger micellar aggregates can form via fractal aggregation of pre‐formed primary micelles, which act as constituent particles that come together through diffusion or reaction‐limited processes.

Upon addition of IPA, the DLS profile showed a clear shift toward larger hydrodynamic sizes, with the primary micelle peak shifting from 32.7 nm to 45.8 nm, indicating that IPA binding induces an increase in micellar number, which refers to the number of CP3 polymer chains per primary micelle. Concurrently, the micellar aggregate peak shifted from 164 nm to 275 nm. To quantify the extent of this IPA‐induced aggregation, we applied a fractal model for spheres [58, 59] that links the size of a micellar aggregate to the number of constituent primary micelles within it, based on (1):

(1) $$R_{Hs}={\left(\frac{n}{k_{f}}\right)}^{1/D_{f}}R_{p}$$

where *k_f_* represents the scaling parameter (1.81) and *D_f_* represents the fractal dimension (2.05) for a reaction‐limited cluster aggregation mechanism. *R_p_* is the radius of the primary micelle and *R_Hs_* is the radius of micellar aggregate. Rearranging the equation, we obtain (2):

(2) $$n=k_{f}{\left(\frac{R_{Hs}}{R_{p}}\right)}^{D_{f}}$$

where *n* is the number of primary micelles that form the micellar aggregate. The shift of the micellar aggregate peak from 164 nm to 275 nm reveals that *n* increased from approximately 49 to 71 primary micelles upon IPA binding. Both resultant bands also became much broader than before, suggesting an increase in particle size heterogeneity post IPA binding. This micellar conformational change is driven by specific molecular interactions between IPA and CP3. The carboxylate group of IPA can form electrostatic interactions with the cationic side chains of CP3, while the indole group of IPA can engage in π‐π stacking with the CP3 polymer backbone. These combined interactions promote inter‐micelle association, which causes agglomeration of the CP3 polymer micelles, consistent with DLS measurements. The IPA‐induced micellar growth is likely the mechanism underlying the selective fluorescence quenching of the CP3 polymer, as the structural reorganization leads to enhanced non‐radiative decay and aggregation‐induced quenching. In contrast, melatonin, which lacks a carboxylate group does not induce a comparable increase in micellar number or aggregation of CP3 micelles. Hence, the addition of melatonin does not result in significant polymer fluorescence quenching.

To quantify the sensitivity of the CP3 polymer's interaction with IPA, we titrated the polymer solution with increasing concentrations of IPA, ranging from 1 µM to 500 µM (Figure 3d) and correlated the resulting fluorescence quenching intensity with the corresponding IPA concentration. Analysis of this titration data using the same Langmuir isotherm model yielded a K_D_ of 32 µM for the IPA‐CP3 interaction, with a LOD for IPA calculated to be 3.15 µM (Figure 3e). These sensitivity values are comparable to the performance of the CP3‐SWNT sensor. Further key in vitro sensor performance parameters, such as linearity, accuracy and precision, have also been derived from the IPA calibration curves of CP3‐SWNT and CP3 polymer (Table S1). With SWNTs generally recognized to be photo‐stable [60], we also assessed the photostability of the CP3 polymer itself under constant photo‐exposure. Under continuous UV‐A irradiation at 350‐nm and 380‐nm, the CP3 polymer's fluorescence remained stable for up to 1 h (Figure 3f). In contrast, under UV‐B photo‐exposure at 280‐nm, CP3 polymer fluorescence was less stable, with significant photobleaching occurring within 16 min. These results suggest that UV‐A excitation wavelengths are preferable for maintaining the sensor's signal stability, and thus, UV‐A excitation (350/380‐nm) was used in subsequent experiments involving patient plasma samples.

### 2.4 CP3‐SWNT Hydrogel Development

**FIGURE 4:**
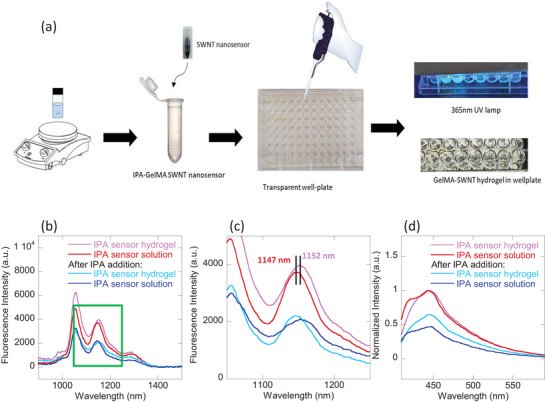
IPA GelMA sensor hydrogel development. (a) Schematic illustrating the synthesis of IPA‐GelMA sensor hydrogel which involves mixing of GelMA and sensor together with Irgacure 2959 photoinitiator, followed by UV‐photo‐crosslinking; (b) NIR fluorescence spectra of CP3 wrapped Comocat (7,6)‐enriched SWNT solution and hydrogel, before and after addition of IPA. Both sensor solution and hydrogel exhibit similar NIR quenching in response to addition of IPA. (c) Zoom‐in of NIR fluorescence spectra marked with green box in Figure 4b showing 5‐nm red‐shift in the (7,6)‐SWNT fluorescence band in GelMA hydrogel matrix compared to solution form. (d) Normalized emission spectra of CP3 polymer solution and hydrogel, before and after addition of IPA. Both sensor solution and hydrogel exhibit similar quenching in the blue region, when IPA is added.

The use of SWNT‐based CoPhMoRe sensors in the NIR region is particularly advantageous due to deeper biological tissue penetration of NIR light with reduced adsorption and scattering, compared to visible light [61, 62]. To create a stable sensor platform, a biocompatible sensor form factor, such as a hydrogel matrix, is essential for localizing the sensor and preventing its diffusion. In previous work, poly(ethylene glycol) diacrylate (PEGDA) was successfully employed as an encapsulating material for an implantable progesterone CoPhMoRe sensor, demonstrating molecular recognition feasibility in vivo after sensor implantation into a murine model [36]. Here, we attempted to synthesize IPA sensor PEGDA hydrogels, but encountered challenges in achieving repeatable photo‐crosslinking of the CP3‐SWNT‐embedded PEGDA hydrogel. Consequently, we chose gelatin methacryloyl (GelMA) as the encapsulating material for the IPA sensor (Figure 4a). GelMA, a modified form of gelatin, is a natural protein that offers excellent biocompatibility and tunable mechanical properties [63, 64]. SWNT‐GelMA hydrogels have also been previously used for nitric oxide sensing and integrated into implantable tags for ex vivo implantation into biological tissues, validating their efficacy in tissue‐relevant environments [65].

Upon generation of the CP3‐SWNT hydrogel, we compared its NIR fluorescence response to IPA addition with that observed in solution phase (Figure 4b). We postulated that the crosslinked hydrogel matrix might reduce IPA sensor sensitivity compared to the solution phase, due to restricted movement and potential reconfiguration of the CP3 polymer corona around the SWNTs. Remarkably, we observed that the GelMA hydrogel‐encapsulated CP3‐SWNT sensor exhibited a slightly higher quenching response to IPA (47%) than the response observed in the solution phase (41%). This enhanced response within the hydrogel could be attributed to a pre‐concentration of the IPA analyte within the hydrogel matrix, leading to increased IPA‐corona interactions. Furthermore, we noticed a 5‐nm red‐shift of the (7,6)‐SWNT fluorescence peak from 1147‐nm in the solution phase to 1152‐nm in the hydrogel (Figure 4c). This shift toward longer wavelength is likely due to slight aggregation or re‐organization of the CP3 polymer corona around the SWNTs within the confined hydrogel environment, potentially impacting analyte binding [66, 67].

### 2.5 CP3 Polymer Hydrogel Development

As a complementary platform for IPA measurements [68], the CP3 polymer alone can also be embedded within the crosslinked GelMA matrix using the same approach without SWNTs (Figure 4d). Polymer fluorescence is retained after hydrogel formation. However, fluorescence quenching upon IPA addition is reduced in the hydrogel‐encapsulated CP3 polymer (35%) compared to the solution phase (53%). Nonetheless, these results demonstrate the feasibility of encapsulating both the CP3‐SWNT and the CP3 polymer within a GelMA hydrogel matrix, while retaining their sensor capabilities. The hydrogel encapsulation approach provides a viable strategy for achieving biocompatibility, consistent with established methodologies for implantable in vivo nanosensors [36, 65, 68]. This sets the foundation for future development of continuous real‐time IPA monitoring.

### 2.6 IPA Sensor Demonstration in Patient Plasma Samples (CP3 Polymer)

Given the enhanced selectivity of the CP3 polymer for IPA over the CP3‐SWNT sensor, the polymer alone was chosen for IPA detection in patient plasma samples. The visible fluorescence of CP3 also allowed for higher throughput measurements using a multi‐well plate reader. This enables rapid analysis of numerous patient samples, in contrast to the lower throughput of NIR fluorescence measurements that typically require individual quartz cuvette measurements.

To investigate the relationship between IPA levels and gut health, plasma samples from 125 participants were categorized into healthy controls, or those with gastrointestinal diseases, such as those with ulcerative colitis, Crohn's disease, colonic tubular adenomas, and colorectal adenocarcinoma (25 participants per category). These patient groups represent distinct gastrointestinal conditions crucial for validating the sensor's clinical relevance. Healthy controls are defined as individuals without any diagnosed gastrointestinal conditions, included to establish a baseline range of normal IPA concentrations. Ulcerative colitis and Crohn's disease are both chronic inflammatory bowel diseases. Ulcerative colitis primarily affects the colon causing continuous and superficial lesions, while Crohn's disease could affect any part of the digestive tract causing skip lesions which are inflamed areas interspersed between healthy tissues [69, 70]. Colorectal adenocarcinoma refers to malignant tumors originating in the colon or rectum, commonly known as colorectal cancer [71], while colonic tubular adenomas denotes benign but potentially pre‐cancerous polyps that develop in the colon or rectum [72, 73]. Patients with intestinal inflammation, specifically those with ulcerative colitis and Crohn's disease are often associated with dysbiosis, an imbalance in the gut microbiota, and altered gut microbial metabolism [74, 75, 76]. Prior HPLC studies have established that IPA levels are significantly reduced in patients with active gut inflammation, such as ulcerative colitis [10]. Therefore, we designed our study to validate the CP3 polymer sensor's capability to detect this trend, by comparing the IPA levels in the inflammation groups (ulcerative colitis and Crohn's disease) against healthy controls. We also investigated if IPA levels were altered in the patients with colonic tubular adenomas and colorectal adenocarcinoma, given previous predictions of the lower abundance of essential amino acids and tryptophan‐associated metabolites in patients with colonic tubular adenomas [77].

**FIGURE 5:**
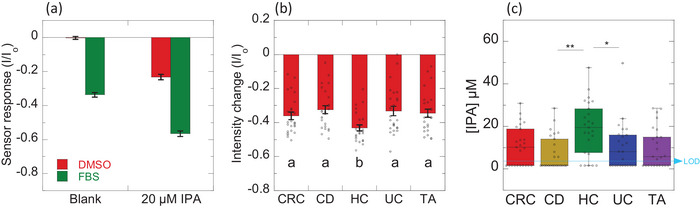
Blue‐fluorescent IPA sensor applied in plasma samples obtained from clinical patients. (a) Sensor response of CP3 polymer, after addition of blank DMSO and blank FBS (left), as well as after addition of 50 µM IPA dissolved in DMSO (red) and 20 µM IPA‐spiked FBS (green). I_o_ is the initial fluorescence intensity of CP3 polymer prior to addition of analytes. Error bars represent standard deviations from independent experiments (n = 3); (b) Sensor response of CP3 polymer, after addition of patient plasma samples. Patients are grouped into healthy controls (HC), ulcerative colitis (UC), Crohn's disease (CD), colonic tubular adenomas (TA), and colorectal adenocarcinoma (CRC). Bar graphs show the mean values with error bars representing standard error from n = 25 individuals of each patient group. Dots represent each data point. Different alphabet letters show significant differences using one‐way ANOVA with the Student‐Newman‐Keuls (SNK) post hoc test at *p* < 0.05; (c) Box plots display the distribution of IPA concentrations for CRC, CD, HC, UC and TA patient groups. IPA concentrations are derived from the sensor calibration curve, using fluorescence intensity of sensor + blank FBS as baseline. The sensor limit of detection (LOD) is 3.15 µM, with signal‐to‐noise ratio = 3 (blue line). Sensor responses that are below LOD are plotted as individual data points at 1.575 µM (LOD/2). Boxes represent the interquartile range (IQR), with the line within each box indicating the median. Whiskers extend to 1.5 times the IQR from the box. Patient groups were compared using the Kruskal‐Wallis test, followed by Dunn's post‐hoc test with Holm correction. Asterisks indicate statistical significance: * *p* < 0.05, ** *p* < 0.01. The IPA sensor reports significant differences in IPA concentration between the HC patient group and patients with active gut inflammations, UC (*p* = 0.04) and CD (*p* = 0.003).

As a preliminary assessment, the sensor's response to IPA was first evaluated in commercially available fetal bovine serum (FBS) medium (Figure 5a). The addition of FBS alone resulted in a 33% quenching of the CP3 polymer's fluorescence. Subsequent spiking of the FBS with 20 µM IPA led to a further 23% fluorescence quenching, resulting in a total quenching of 56%. In comparison, the addition of blank DMSO did not induce significant fluorescence quenching, whereas subsequent addition of 20 µM IPA elicited a comparable 23% fluorescence quenching. These findings indicate that despite the initial fluorescence quenching caused by the FBS medium, the CP3 polymer sensor retains its functionality in this biological matrix. Though the visible channel was preferable for high‐throughput testing of plasma samples, we have also verified that the CP3‐SWNT sensor remains functional using IPA‐spiked FBS (Figure S6). We then verified that the CP3 polymer sensor shows a concentration‐dependent quenching response that increases with concentration of IPA spiked in human plasma samples (Figure S7a). The increasing trend aligns closely with LC‐MS analysis of IPA‐spiked human plasma and FBS samples (Figure S7b,c), validating the reliability of the sensor's response in complex biological matrices.

We then proceeded to measure the IPA sensor response to addition of plasma samples from the different patient groups (Figure S8) using the CP3 polymer sensor. As observed in Figure 5b, the sensor's fluorescence quenching had significant variability among individuals, even within the same group. This variability could be attributed to inherent differences in IPA levels, or variations in the complex biological plasma environment. The average quenching intensity for the patient groups of Crohn's disease and ulcerative colitis fell within a similar range (32%–33%) to that of the blank FBS, suggesting relatively low IPA levels in these groups. In contrast, the colorectal adenocarcinoma and colonic tubular adenoma groups showed higher average quenching intensities of 36% and 35% respectively. Notably, the healthy controls group displayed a statistically significant increase in average fluorescence quenching (43%), indicative of higher IPA levels compared to the other patient groups. These observations align with previous research demonstrating depleted IPA levels in individuals with active gut inflammations, such as ulcerative colitis [10].

To estimate the IPA concentrations in patient samples with the nanosensor, we applied the CP3 polymer IPA calibration curve, using the quenching percentage of blank FBS serum as a baseline. It is important to note that while blank FBS served as a baseline, a subset of patient samples exhibited quenching intensities at or below the sensor's detection limit (LOD) of 3.15 µM. This seemingly lower quenching could be attributed to the inherent variability in the complex background matrix of patient plasma samples, which may differ significantly from the FBS matrix. The variability in background quenching could then result in some plasma samples showing a net quenching signal lower than the FBS baseline. Nonetheless, for these samples falling below LOD, IPA concentrations were conservatively estimated as LOD/2. Analysis of the estimated IPA concentrations using the Kruskal‐Wallis test revealed significant differences among the groups. Specifically, post hoc analysis with Dunn's test showed that the median IPA concentration of the healthy controls group (19.56 µM) was significantly higher than that of the ulcerative colitis group (8.17 µM) (*p* = 0.043, Holm‐corrected). Notably, the Crohn's disease group had a majority of data points below the sensor's detection limit. While the sensor‐derived median IPA concentration of the healthy controls group was also higher than those of the colorectal adenocarcinoma (10.30 µM) and colonic tubular adenomas (5.79 µM) patient groups, these differences did not reach statistical significance in the post hoc analysis. This lack of statistical significance is likely due to the substantial spread in IPA concentrations observed among individual patients within the patient groups.

Therefore, the IPA sensor has successfully captured a trend of reduced IPA concentration in the Crohn's disease and ulcerative colitis groups compared to the healthy controls group, which demonstrates the sensor's performance in a relevant clinical context, aligning with established studies that gut dysbiosis negatively impacts IPA production by gut bacteria. However, the sensor shows limitations in sensitivity, as evidenced by the number of patient samples falling below the sensor's detection limit. Furthermore, the considerable overlap in the distribution of IPA concentrations across different patient groups poses a challenge for accurate classification of individual samples and reliable prediction of gut health based solely on IPA sensor readouts. Despite these limitations, the successful demonstration of the IPA sensor offers a valuable complement to existing clinical features for gut health monitoring and paves the way forward for future gut health monitoring strategies employing multiplexed sensors for detection of key indole‐based gut metabolites.

### 2.7 Conclusions and Future Work

In summary, this study demonstrates the successful development of two complementary optical nanosensor platforms for IPA detection, which exhibits selective fluorescence quenching responses to IPA in the visible (CP3 polymeric micelles) and NIR (CP3‐SWNT) ranges. We have shown that CP3 self‐assembles into blue‐fluorescent polymeric micelles in aqueous solution, forming one of the sensing channels. Non‐covalent wrapping of SWNTs with CP3 creates the second NIR‐responsive channel, which exhibits photophysical behavior suggestive of FRET. In both channels, the sensor's response to IPA is conserved when encapsulated within a biocompatible GelMA hydrogel matrix, establishing a foundation for future in vivo applications that require continuous tracking of IPA levels. To fully realize the in vivo potential of the sensor hydrogel, future work will include comprehensively validating its biocompatibility through in vitro cytotoxicity assays and in vivo implantation studies [68]. Through modifications to the hydrogel's physical properties, such as swelling kinetics, cross‐linking density, and porosity, we aim to minimize potential nanomaterial toxicity and adverse tissue response, while maintaining optimal analyte diffusion and binding. Long‐term performance and stability of the encapsulated sensor will also be evaluated under physiological conditions. While our current study focuses on plasma samples, IPA levels in tissues can provide critical information about localized gut inflammation, which may complement systemic information obtained from blood. The optimized sensor hydrogel can therefore be used to measure IPA levels directly in biopsy tissue samples, enabling a more rapid and localized assessment of gut inflammation status.

Application of the sensor to patient plasma samples captured a statistically significant trend of reduced IPA levels in patient groups with active gut inflammations, Crohn's disease and ulcerative colitis, compared to healthy controls. This is consistent with prior literature that IPA levels in these patients would be significantly lower as inflammation can disrupt production of IPA by gut bacteria. However, due to inherent variability in patient IPA levels and limitations in sensor's sensitivity, comparisons of IPA levels in cancer patient groups (colorectal adenocarcinoma and colonic tubular adenomas) against healthy controls lacked statistical significance. Hence, accurate classification of gut health status solely based on IPA sensor readouts remain challenging. To improve diagnostic accuracy, future work will focus on several key areas. First, we will explore sensor multiplexing for simultaneous detection of a panel of key gut metabolites, such as IAA and Trp, to facilitate a more comprehensive gut health assessment. Second, we will investigate approaches to enhance the nanosensor's detection limit, which currently remains several orders of magnitude higher than state‐of‐the‐art LC‐MS methods that achieve limits of quantification in the nM range (3–285 nM) [78, 79]. Through hardware design and innovation, such as integrating an amplified photodetector, we aim to enhance the signal‐to‐noise ratio of the optical sensor to extend its sensitivity below the current µM range, advancing its potential as a point‐of‐care diagnostic platform complementary to LC‐MS. Finally, to enhance the clinical applicability of the sensor, our future efforts will focus on strategies to optimize its bio‐interface with complex human samples to minimize non‐specific sensor fluorescence quenching. These strategies include confining of the nanosensors within biocompatible microneedles and integrating of the nanosensors in microfluidic platforms. The combination of these advancements will culminate in the development of a promising point‐of‐care sensing device that will transform gut health diagnostics and analysis, enabling more proactive and personalized management of gastrointestinal health.

## 3 Experimental Section

### 3.1 Materials

All reagents, monomers, bio‐analytes and solvents were purchased from Sigma‐Aldrich Ltd, unless otherwise stated. Purified HiPco SWNTs were purchased from NanoIntegris, while Comocat (7,6) enriched SWNTs were purchased from Sigma‐Aldrich Ltd. Both SWNTs were used without further processing or purification.

### 3.2 Synthesis of CP3 Polymer

2,5‐dibromopyrimidine (Pm‐diBr) and 2,7‐Dibromo‐9,9‐bis[3‐(dimethylamino)propyl]fluorene (F‐diBr) monomers were purchased from Tokyo Chemical Industry Co. Ltd. To enable the Suzuki‐Miyaura polymerization, the dibromo groups of F‐diBr were converted to diboronic ester (diEs) groups based on previously established procedures, to form 9,9‐bis[3‐(dimethylamino)propyl]fluorene‐2,7‐diboronic acid bis(pinacol) ester (F‐diEs) [31, 80]. For Suzuki‐Miyaura polymerization, the Pm‐diBr and F‐diEs monomers were then mixed at equimolar concentrations in a crimp‐sealed microwave vial, together with 3 mol% palladium catalyst: [1,1′‐bis(diphenylphosphino)ferrocene]‐dichloropalladium(II) [Pd(dppf)Cl_2_]. The mixture was dissolved in degassed anhydrous THF: DMF (2:1) solvent mixture, followed by addition of degassed base Na_2_CO_3_ (5 molar equivalents). The reaction mixture was then subjected to heating in a Biotage Initiator+ microwave reaction chamber at 130°C for 15 min. Upon cooling to room temperature, the mixture was precipitated in water and the polymer solids were retrieved by centrifugation. Post‐centrifugation, the solution was decanted and the polymer solids were washed with water 3 times to remove the excess Na_2_CO_3_ base. The polymer solids were then redissolved in THF through a 0.45‐µm nylon syringe filter to remove other insoluble impurities. To convert the polymer to a cationic form, the polymer solids (100 mg) were first dissolved in THF (2 mL), followed by addition of excess 1 m HCl (3 mL). Upon addition of HCl, the polymer solution turned cloudy and re‐dissolved when deionized water (5 mL) was added. The dissolved cationic polymer solution was filtered through a 0.45 µm nylon syringe filter again before transferring to a Spectra‐Por Float‐A‐Lyzer G2 dialysis tube (3.5‐5 kDa MWCO). The cationic polymer solution was dialyzed with ultrapure water for 2 days with the external dialysate refreshed after every 9 h, ensuring removal of excess HCl and other impurities such as the Pd catalyst residues. Finally, post dialysis, purified CP3 polymer solids were retrieved via freeze‐drying for ^1^H NMR analysis (Figure S9) in Methanol‐d4 solvent. CP3 polymer molecular weight (M_n_) was also calculated by ^1^H NMR end‐group analysis to be 25.8 kDa.

### 3.3 Suspension of SWNTs Using CP3 Polymer

For the preparation of the IPA sensor CP3‐SWNT, 5 mg of CP3 polymer was dissolved in ultrapure water to a final concentration of 2.5 mg/mL, followed by the addition of 2 mg of HiPco SWNT or Comocat (7,6) SWNT at a concentration of 1 mg/mL. The mixture was sonicated in an ice bath, with a 3‐mm probe tip (Qsonica Q500) for 30 min at 25% amplitude. The CP3‐wrapped SWNT suspension was diluted to 4 mL and ultra‐centrifuged at 153,100 g for 2 h, to remove excess SWNT aggregates. Upon ultra‐centrifugation, the supernatant was collected and concentration of CP3‐SWNT suspensions were determined by UV–Vis‐NIR spectrophotometry (Agilent Cary 5000), using SWNT absorbance values at 632 nm with 0.036 (mg/L)^−1^ cm^−1^ as the SWNT extinction coefficient [45].

### 3.4 IPA Sensor GelMA Hydrogel Synthesis

For the preparation of IPA‐GelMA hydrogel, 50 mg of GelMA was dissolved in 5 mL of ultrapure water in a glass vial to achieve a final concentration of 10 mg/mL. Then, 5 mg of Irgacure 2959 photo‐initiator was added to the same vial to achieve 1 mg/mL final concentration. The mixture was stirred with a magnetic stirrer, and heated to 40°C for 15mins until fully dissolved. Subsequently, 5 mL of CP3 polymer (0.4 mg/mL) or CP3 SWNT (10 mg/L) was added to the GelMA solution and gently mixed with a pipette to prevent bubble formation. The glass vial was wrapped with aluminium foil to prevent premature degradation and crosslinking. The final CP3‐GelMA or CP3‐SWNT‐GelMA mixture was pipetted into 96‐well plates (100 µL in each well) and exposed to 365‐nm light for around 5 min to trigger UV crosslinking.

### 3.5 IPA Sensor (CP3‐SWNT) Selectivity Screening in the NIR Region

Selectivity screening was conducted using Horiba Flurolog‐3 spectrofluorometer for the NIR channel. CP3‐SWNT was diluted to 5 mg/L in ultrapure water. 990 µL of the diluted SWNT solution was added to the quartz cuvette for measurement of initial SWNT fluorescence prior to addition of analytes. Analytes were each prepared at 10 mM in DMSO and 10 µL of each analyte was separately added to diluted SWNT solutions to achieve a final analyte concentration of 100 µM. Upon addition of analyte, SWNT solutions were gently stirred to ensure mixing. Samples were excited at 650 nm wavelength with a band‐width of ±25 nm while NIR fluorescence emission was collected from 900–1500 nm. Integration time of each scan was 30 s. For 2D excitation‐emission maps, excitation wavelengths were tuned at 5‐nm steps from 300–800 nm, to generate SWNT fluorescence spectra (900–1500 nm) at each step (Integration time = 30 s). As such, each excitation‐emission map took about 1.5 h to acquire. Upon IPA addition, fluorescence quenching occurred rapidly (within ∼1 min) after IPA addition and remained consistent throughout the ∼1.5 h acquisition period, indicating a stable sensor response. For CP3‐SWNT GelMA hydrogel, 1 mL of the sensor hydrogel was prepared and UV‐crosslinked (365 nm) directly in the quartz cuvette. Thereafter, SWNT fluorescence spectra of the hydrogel was taken prior to IPA addition. After addition of 10 µL of IPA at 10 mM to the hydrogel, SWNT fluorescence spectra was taken again after 1 min. Intensity change upon addition of IPA was calculated based on integration of SWNT fluorescence across 900–1400 nm, to account for all the major SWNT chiralities.

### 3.6 IPA Sensor (CP3 polymer) Selectivity Screening in the Visible Region

Selectivity screening was conducted using Varioskan LUX Microplate for the visible channel. For CP3 polymer fluorescence measurement in solution, the polymer was first diluted to 0.4 mg/mL in ultrapure water, and 100 µL of the diluted polymer solution was pipetted into each well. Initial polymer fluorescence was measured before addition of analytes. Analytes were each prepared at 2.5 mM in DMSO, and 4 µL of each analyte was separately added to diluted CP3 solution wells, to achieve a final analyte concentration of 100 µM. To ensure mixing of analyte with polymer solution, the well plate was subjected to shaking for 1 min, prior to measurement. Samples were excited at either 350 nm or 380 nm, with polymer emission spectra collected from 400–600 nm. Each analyte was added to 3 wells to obtain sensor data in triplicates. For the CP3‐GelMA hydrogel, UV photo‐crosslinking occurred directly in the 96 well plates (100 µL in each well). Similarly, polymer fluorescence was measured before and after addition of 100 µM IPA. For calculation of fluorescence intensity change upon addition of IPA, polymer emission was integrated across 400–600 nm.

### 3.7 CP3 Polymer Photostability characterization

To evaluate the photostability of the CP3 polymer, the polymer was diluted to a final concentration of 0.4 mg/mL and 100 uL of the diluted polymer was pipetted into separate wells of the microplate. The well plate was continuously exposed to either 280, 350 and 380 nm excitation for 1 h, while fluorescence signal was measured every 4 min in order to assess fluorescence signal decay over time. The CP3 fluorescence peak at 445‐nm for each measurement was extracted and plotted against the time to evaluate the photostability of CP3 polymer in the visible channel.

### 3.8 IPA Screening of Human Plasma Samples

A total of 125 human plasma samples from 5 different patient groups: healthy controls, ulcerative colitis, Crohn's disease, colonic tubular adenomas, and colorectal adenocarcinoma (25 individuals per group), were obtained from National University Hospital (NUH) for assessment of IPA sensor clinical relevance. The plasma samples were thawed and mixed thoroughly with pipetting before use. For each measurement, the initial fluorescence of IPA sensor (0.4 mg/mL, 100 µL) in 96 well‐plate was measured under 350‐nm excitation. Subsequently, 4 µL of each plasma sample was added to each well. After shaking the well‐plate for 1 min, fluorescence signal of the sensor was measured again at 400—600 nm. Each plasma sample measurement was conducted in triplicates and average fluorescence intensity change (I/I_o_) of each sample was calculated. Statistical analysis was carried out using MATLAB R2024a. For analysis of sensor readouts, one‐way ANOVA with the Student‐Newman‐Keuls (SNK) post hoc test was carried out to determine significance, defined as p ≤ 0.05. For analysis of sensor‐derived IPA concentrations in plasma samples, the Kruskal‐Wallis test (H(4) = 14.733, p = 0.005), followed by Dunn's post‐hoc test with Holm correction, was carried out to determine significance. The IPA sensor evaluation in human plasma samples were performed in accordance with the Research Compliance Policy on Human Subject and Biomedical Research and approved by the Institutional Review Board (IRB) at National University of Singapore (IRB approval number: NUS‐IRB‐2021‐319). Informed consent was obtained from human participants of this study.

### 3.9 LC‐MS Measurement of IPA

IPA standards were prepared by serial dilution to obtain concentrations of 5, 10, 20, 40, 60 µM using FBS as diluent, and concentrations of 5, 20, 50 and 100 µM using human plasma as diluent. Stable isotope labelled internal standard containing Tyrosine‐(phenyl‐d4) was added to each aliquot of 30 µL of standard. The mixture was dried down, derivatized using phenyl isothiocyanate by incubating at room temperature for 1 h, then reconstituted in 5 mM ammonium acetate in methanol. After centrifugation to pellet the precipitated protein, supernatant of samples were diluted with water then analysed using LC‐MS. Analysis was carried out on a Waters Acquity UPLC BEH C18 column (1.7 µm, 2.1×50 mm) using a Waters Acquity I‐Class liquid chromatography system coupled to a Waters Xevo TQ‐XS mass spectrometer. The LC run was performed using 0.2% formic acid in water as mobile phase A and 0.2% formic acid in acetonitrile as mobile phase B, starting with initial gradient of 5% B then increasing to 12% B at 1.5 min, 17.5% B at 2.7 min, 50% B at 4 min, 100% B at 4.5–5.0 min, before returning to 5% B from 5.1‐5.8 min. The flow rate was held at 0.8 mL/min except during 4.7–5.1 min where it was raised to 1.0 mL/min. The column temperature was set at 50°C, and the injection volume was 5 µL. The transition used for IPA was 190> 130. The cone voltage was 40 V, and the collision energy was 14 eV. The transition used for tyrosine internal standard was 321> 140. The cone voltage was 26 V, and the collision energy was 45 eV. All compounds were ionized in positive mode using electrospray ionization. LC‐MS data was processed using Waters TargetLynx software v4.2. The standard curve for IPA quantification was generated by plotting normalized response (Area/Internal Standard Area) against the nominal concentration of the IPA standards.

### 3.10 Statistical Analysis

All statistical analysis was carried out using either MATLAB R2024a or Kaleidagraph Version 4.5. All quantitative data in bar charts are presented as the mean, with error bars representing either the standard deviation or standard error from independent samples as stated in the corresponding figure legends. For box plots, the boxes represent the interquartile range (IQR), with the line within each box indicating the median, and whiskers extending to 1.5 times of IQR. For calculation of sensor intensity change (I/I_o_), the CP3 polymer visible emission was integrated across 400–600 nm while the CP3‐SWNT NIR emission was integrated across 900–1300 nm. Difference in the integrated fluorescence intensity before and after addition of samples defined the sensor response. To derive IPA concentrations from sensor readouts, the established sensor calibration curve correlating I/I_o_ to IPA concentration was applied. If the derived concentration of the sample falls below the sensor's LOD, the concentration was estimated as LOD/2 for statistical analysis. No other data transformation or outlier removal was performed. Sample sizes (n) for all experiments are reported in the corresponding figure captions. For analysis of sensor readouts in patient plasma samples, one‐way ANOVA with the SNK post hoc test was carried out to determine significance, defined at (p ≤ 0.05). For analysis of sensor‐derived concentrations in patient plasma samples, the non‐parametric Kruskal‐Wallis test followed by Dunn's post‐hoc test with Holm correction for multiple comparisons, was utilized to determine significance, also defined at (p ≤ 0.05).

## Conflicts of Interest

The authors declare no conflict of interest.

## Supporting information




**Supporting File**: adhm71092‐sup‐0001‐SuppMat.docx.

## Data Availability

The data that support the findings of this study are available from the corresponding author upon reasonable request.
