# Supplementary material for: Fluorescent Nanosensor for Indole‐3‐Propionic Acid Detection in Gut Health Monitoring
Source: Adv Healthc Mater. 2026 May 2;15(21):e03434. doi: 10.1002/adhm.202503434 (PMC13241470; doi:10.1002/adhm.202503434)
Supplement: Supplementary file 1 — Supporting File: adhm71092‐sup‐0001‐SuppMat.docx. [file ADHM-15-0-s001.docx]

**Supporting Information**

**Fluorescent Nanosensor for Indole-3-propionic Acid Detection in Gut Health Monitoring**

Mervin Chun-Yi Ang^1,2#^, Jonathan Wei Jie Lee^3,4,5#^, Sayyid Mohaideen^1^, Gabriel Sánchez-Velázquez^6^, Song Wang^1,7^, Yangyang Han^1^, Liu Lin^3^, Germaine Yong^8^, Raju Cheerlavancha^1^, Duc Thinh Khong^1^, Jianhong Ching^9^, Sharon Hong Yu Han^9^, Jun Yu Yeo^1^, Lian Xu^1^, Gajendra Pratap Singh^1^, Michael S. Strano^1,6*^

^1^Disruptive & Sustainable Technologies for Agricultural Precision IRG, Singapore-MIT Alliance for Research and Technology, 1 CREATE Way, #03-06/07/08 Research Wing, Singapore 138602, Singapore

^2^Natural Sciences and Science Education, National Institute of Education, Nanyang Technological University, 1 Nanyang Walk, Singapore 637616, Singapore

^3^Yong Loo Lin School of Medicine, National University of Singapore, 10 Medical Drive, Singapore 117597, Singapore

^4^Division of Gastroenterology and Hepatology, National University Hospital, 5 Lower Kent Ridge Rd, Singapore 119074, Singapore

^5^iHealthtech, National University of Singapore, 14 Medical Dr, #14-01 MD6, Singapore 117599, Singapore

^6^Department of Chemical Engineering, Massachusetts Institute of Technology, 77 Massachusetts Avenue, Cambridge, MA 02139, USA

^7^State Key Laboratory of Mesoscience and Engineering, Institute of Process Engineering, Chinese Academy of Sciences, 1 North 2nd Street, Zhongguancun, Haidian District, Beijing 100190, China

^8^Singapore Institute of Food and Biotechnology Innovation (SIFBI), Agency for Science, Technology and Research (A*STAR), 31 Biopolis Wy, #04-01 Nanos, Singapore 138669, Singapore

^9^Cardiovascular and Metabolic Diseases Programme, Duke-National University of Singapore Graduate Medical School, 8 College Rd, Singapore 169857, Singapore

*Corresponding authors’ email address: [strano@mit.edu](mailto:strano@mit.edu)

^#^ These authors contributed equally to this work.

**SUPPLEMENTARY TEXT**

**Molecular Probe Adsorption (MPA) method**

The MPA method quantifies the accessible nanoparticle surface area (i.e., exposed carbon surface) of the CP3-SWNT construct, providing insight into the compactness of the corona phase and its availability for analyte binding. It uses riboflavin, a fluorescent dye (λ_max_ = 532 nm), as a probe to characterize the SWNT surface coverage. Riboflavin can exist in free or SWNT-bound states; free riboflavin retains its intrinsic fluorescence, while bound riboflavin exhibits quenched fluorescence. Its isoalloxazine ring enables strong π-π interactions with SWNTs, contributing to its high binding affinity. The corona phase shields the SWNT surface from riboflavin binding, so the magnitude of riboflavin fluorescence quenching correlates directly with the exposed SWNT surface area.

The Langmuir isotherm is applied to model the riboflavin-SWNT interaction:

$C_{total}= C_{ribo}+qC_{NP}\frac{C_{ribo}}{C_{ribo}+K_{D}}$ (1)

where $C_{total}$ represents the total concentration of riboflavin added, $C_{ribo}$ represents the concentration of unbound riboflavin, and $C_{NP}$ represents the nanoparticle concentration. *q* is a dimensionless quantity representing the maximum amount of probe that can adsorb per mole of nanoparticle. It reflects the accessible surface area of the SWNT and the extent of surface coverage by the corona phase. $K_{D}$ represents the dissociation constant of the riboflavin probe and SWNT binding.

The CP3-SWNT solution typically contains excess free CP3 polymer, which can also bind to riboflavin and contribute to fluorescence quenching independently of the nanotube surface. To account for this, an additional background term ($C_{bg}$) is added to (1), resulting in:

$C_{total}= C_{ribo}+q_{NP}C_{NP}\frac{C_{ribo}}{C_{ribo}+K_{D,NP}}+q_{bg}C_{bg}\frac{C_{ribo}}{C_{ribo}+K_{D,bg}}$ (2)

Here, $q_{bg}$ is the number of accessible vacant sites for riboflavin binding with the CP3 polymer, $K_{D,bg}$ is the dissociation constant of riboflavin binding with CP3, and $C_{bg}$ is the concentration of free CP3 polymer. If the free CP3 polymer is removed from the CP3-SWNT solution, $C_{bg}$ approaches zero, and equation (2) reduces to (1). Hence, MPA requires generation of three calibration curves of riboflavin fluorescence versus riboflavin concentration: one in pure aqueous solution, one in CP3 polymer-only solution, and one in CP3-SWNT solution containing excess CP3 free polymer (Supplementary Figure 4a). Strong π-π interactions between the riboflavin isoalloxazine ring and the conjugated polymer backbone of CP3 result in significant riboflavin quenching in the CP3-only solution. Significant quenching is also observed in the CP3-SWNT solution. However, the quenching due to CP3-SWNT alone is relatively small after accounting for background quenching by free CP3. This indicates that the SWNT surface has high coverage by the CP3 corona, with few accessible sites for riboflavin binding.

Rearranging (2), we can derive the key governing equations of MPA:

${\Delta= C}_{total}-C_{ribo}-q_{bg}C_{bg}\frac{C_{ribo}}{C_{ribo}+K_{D,bg}}= q_{NP}C_{NP}\frac{C_{ribo}}{C_{ribo}+K_{D,NP}}$ (3)

$\frac{C_{NP}}{\Delta}=\frac{1}{q_{NP}}+\frac{K_{D,NP}}{q_{NP}}\frac{1}{C_{ribo}}$ (4)

where the slope of (4) is the inverse of $\frac{q}{K_{D}}$ representing the ratio of CP3-SWNT surface area to the riboflavin dissociation constant (Supplementary Figure 4b), which directly corresponds to surface coverage of CP3-SWNT. A high slope (3254.5 μM) corresponds to a small $\frac{q}{K_{D}}$ (307 M^-1^), indicative of a tightly packed corona with limited SWNT surface area available for riboflavin binding.

**Supplementary Figures and Tables with captions**

**Supplementary Figure 1:** Polymerization synthetic scheme for CP3 polymer

**Supplementary Figure 2:** Hydrodynamic size distribution of CP3-SWNT using maximum a posteriori nanoparticle tracking analysis (MApNTA), that shows average corona phase hydrodynamic radius of ~43 nm.


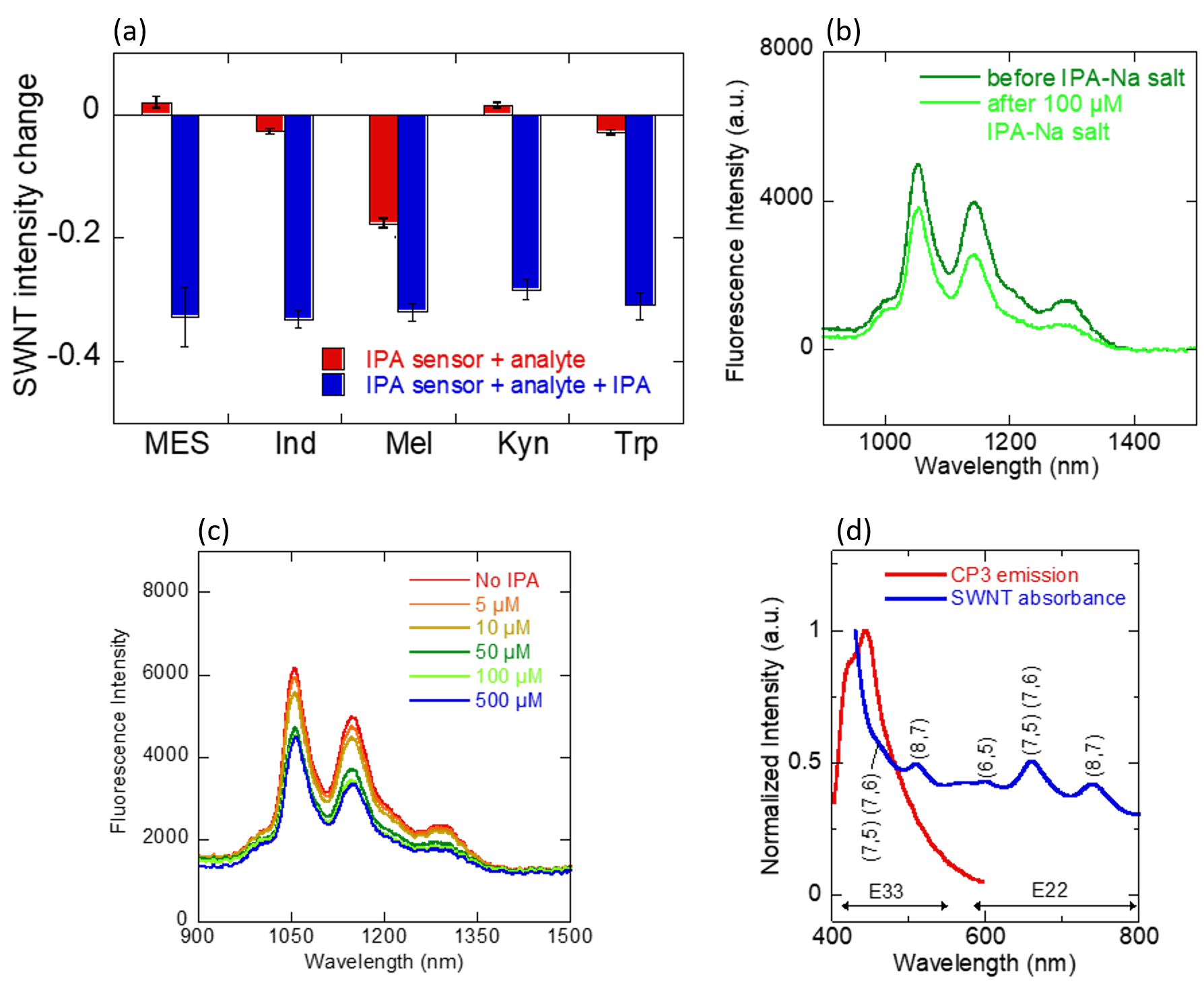


**Supplementary Figure 3:** (a) Fluorescence response of CP3-SWNT in response to 100 μM of interferent gut metabolites before and after addition of 100 μM of IPA. CP3-SWNT exhibits preferential binding affinity to IPA, giving consistent quenching response of >30% in the presence of other gut metabolites; (b) CP3-SWNT fluorescence spectra before (dark green) and after (light green) addition of 100 µM indole-3-propionate sodium salt (IPA-Na); (c) NIR fluorescence spectra of CP3 polymer upon addition of different concentrations of IPA from 5– 500 μM; (d) Emission spectrum of CP3 polymer (red) and UV-Vis absorbance spectrum of CP3 wrapped Comocat (7,6)-SWNT (blue) showing spectra overlap between CP3 emission and CP3-SWNT E33 absorption bands. E33 and E22 absorption bands of different SWNT chiralities are labelled in parenthesis.

**Supplementary Figure 4: Molecular Probe Adsorption (MPA) Method** (a) Linear fittings of riboflavin fluorescence intensity change with addition of 0—5 μM riboflavin in DI water control (red), 5 mg/L CP3 polymer (green) and CP3-SWNT (blue). (b) Linear fitting of $\frac{C_{SWNT}}{\Delta}$ against $\frac{1}{C_{ribo}}$ plot for CP3-SWNT. Inverse of the slope represents $\frac{q}{K_{D}}$, a measure of surface coverage of CP3 polymer on SWNT surface. Smaller $\frac{q}{K_{D}}$ value is indicative of tighter wrapping with less accessible surface area for riboflavin probe adsorption. $\frac{q}{K_{D}}$ of CP3-SWNT = 307 M^-1^.

**Supplementary Figure 5:** (a) Scanning electron microscopy (SEM) image of CP3 polymer micelles. (b) Size distribution of CP3 polymer micelles without SWNTs measured with dynamic light scattering (DLS) before (red) and after addition of IPA (green) or melatonin (blue).


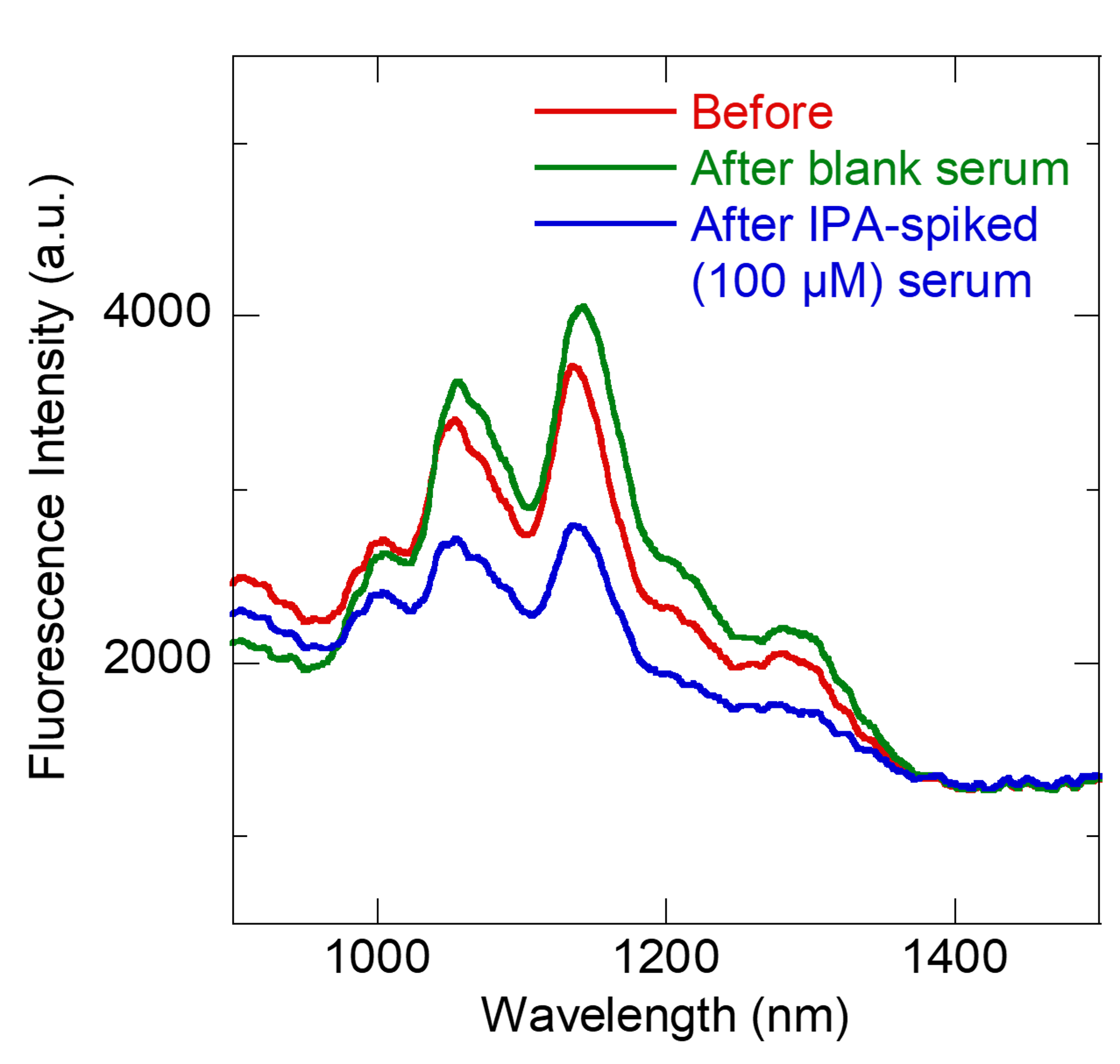


**Supplementary Figure 6:** NIR fluorescence of CP3-SWNT solution before (red) and after mixing with either blank serum (green) and 100 μM IPA-spiked serum (blue), which shows significant quenching response to IPA binding. Excitation wavelength: 785 nm.


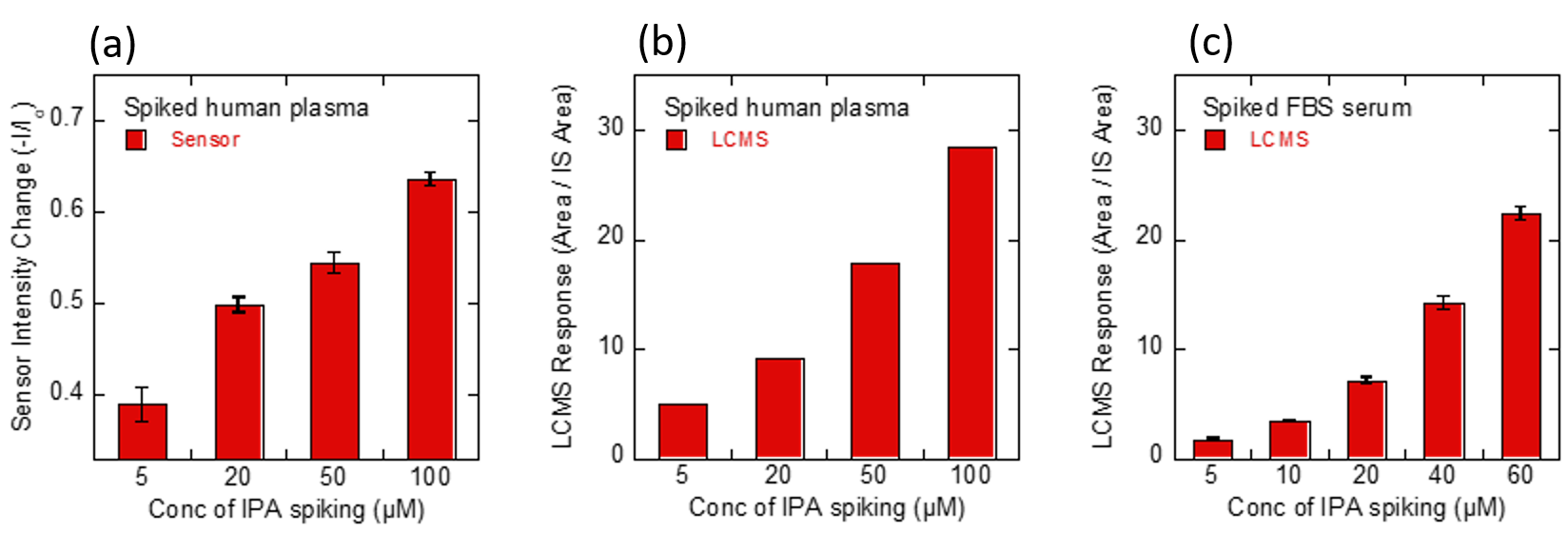


**Supplementary Figure 7:** Validation of IPA quantification in biological matrices (a) Sensor response
(-I/I_o_) to IPA-spiked human plasma samples (n=3) at 5, 20, 50 and 100 µM. (b) LC-MS response (Area / Internal Standard (IS) Area) of IPA-spiked human plasma samples (n=1) at 5, 20, 50 and 100 µM. (c) LC-MS response (Area / Internal Standard (IS) Area) of IPA-spiked FBS (n=6) at 5, 10, 20, 40 and 60 µM.

**Supplementary Figure 8:** Average IPA sensor fluorescence spectra before (red) and after (blue) addition of patient plasma samples. Patients are grouped into healthy controls (HC), ulcerative colitis (UC), Crohn’s disease (CD), colonic tubular adenomas (TA), and colorectal adenocarcinoma (CRC). Shaded regions represent standard error across n = 25 independent individuals.

**Supplementary Figure 9:**  ^1^H NMR of purified CP3 polymer in MeOD solvent.

|  | **NIR channel (CP3-SWNT)** | **Visible channel (CP3 polymer)** |
| --- | --- | --- |
| **Limit of Detection (µM)** | 4.10 | 3.15 |
| **Linearity (R^2^)** | 0.995 | 0.987 |
| **Binding Affinity (K_D_) (µM)** | 18 | 32 |
| **Accuracy (Recovery %)** | 96.5 – 104.5 | 86.3 – 108.9 |
| **Precision (s.d.)** | 0.02 – 0.05 | 0.01 – 0.03 |

**Supplementary Table 1:**  Key IPA sensor performance parameters.
